# ConceptDrift: leveraging spatial, temporal and semantic evolution of biomedical concepts for hypothesis generation

**DOI:** 10.1093/bioinformatics/btaf563

**Published:** 2025-10-28

**Authors:** Amir Hassan Shariatmadari, Alireza Jafari, Sikun Guo, Sneha Srinivasan, Nathan C Sheffield, Aidong Zhang, Kishlay Jha

**Affiliations:** Department of Computer Science, University of Virginia, Charlottesville, VA 22903, United States; Department of Computer Science, University of Virginia, Charlottesville, VA 22903, United States; Department of Computer Science, University of Virginia, Charlottesville, VA 22903, United States; Department of Computer Science, University of Virginia, Charlottesville, VA 22903, United States; Department of Genome Sciences, University of Virginia, Charlottesville, VA 22908, United States; Department of Computer Science, University of Virginia, Charlottesville, VA 22903, United States; Department of Electrical and Computer Engineering, University of Iowa, Iowa City, IA 52242, United States

## Abstract

**Motivation:**

Hypothesis generation is a fundamental problem in biomedical text mining that aims to generate ideas that are new, interesting, and plausible by discovering unexplored links between biomedical concepts. Despite significant advances made by existing approaches, they do not fully leverage the evolutionary properties of biomedical concepts. This is limiting because scientific knowledge continually evolves over time, with new facts being added and old ones becoming obsolete. Thus, it is crucial to capture the evolutionary properties of biomedical concepts from multiple perspectives (e.g. spatial, temporal, and semantic) to generate hypotheses that reflect the up-to-date information landscape of the biomedical domain.

**Results:**

We introduce a novel framework, *ConceptDrift*, that models the hypothesis generation task as a sequence of temporal graphlets and simultaneously encodes spatial, temporal, and semantic change. Unlike existing approaches that treat these dimensions independently, *ConceptDrift* is the first to provide a holistic understanding of concept evolution by integrating them into a unified framework. Grounded in the theories of the Distributional Hypothesis and Conceptual Change, our method adapts these principles to the unique challenges of large-scale biomedical literature. We conduct extensive experiments across multiple datasets and demonstrate that *ConceptDrift* consistently outperforms state-of-the-art baselines in generating accurate and meaningful hypotheses. Our framework shows immediate practical benefits for web-based literature mining tools in life sciences and biomedicine, offering more robust and predictive feature representations.

**Availability and implementation:**

https://github.com/amir-hassan25/ConceptDrift (DOI: 10.6084/m9.figshare.29975476).

## 1 Introduction

Scientific repositories such as PubMed (Jin *et al.* 2023) host more than 35 million articles and add around 3000 articles daily (Lee *et al.* 2020). While this swift availability of scientific information has acted as an impetus for rapid innovation, it has also overwhelmed researchers trying to survey published studies and construct novel ideas. For example, consider a researcher whose objective is to generate/validate a new hypothesis for the treatment of *Parkinson disease*. A PubMed query such as “treatment of Parkinson disease” retrieves more than 80 000 articles. Manually reviewing the literature of this size is impractical for individual researches, and thus it posits a bottleneck to their scientific productivity. To address the above challenge, it is imperative to develop novel computational approaches that can read, reason over the large-scale scientific corpus and help biomedical researchers in forging new, interesting and likely to be true hypotheses for possible *in-vitro* clinical trials. Central to this is the problem of biomedical hypothesis generation (HG) (Akujuobi *et al.* 2020a, Zhao *et al.* 2021, Zhou *et al.* 2024b) that aims to discover cross-silo connections between two disjoint biomedical concepts by chaining together the already known and established scientific facts that remain dispersed across the disparate research fields.

Specifically, given an input concept of interest (e.g. disease or gene), HG aims to find implicit connections (e.g. potential drug target or novel indicator of disease mechanism) that link them in a previously unknown but semantically meaningful way. As an illustration, consider the example of *Raynaud’s disease* and *Fish Oils* discussed in the HG literature (Akujuobi *et al.* 2020a, Zhou *et al.* 2024a, 2022). Prior to 1985, there was no direct connection known between Raynaud’s disease and Fish Oils. However, in 1986, after manually inspecting the titles of articles on both topics separately, researchers inferred and later clinically validated (Swanson, 1986) an association between them. Finding such meaningful implicit links is the crux of the problem that this paper attempts to address.

Over the past decade, approaches ranging from distributional statistics (Srinivasan 2004, Spangler *et al.* 2014), graph theoretic measures (Pu *et al.* 2023, Cameron *et al.* 2015), embedding-based methods (Jha *et al.* 2018, Wang *et al.* 2021a) to most recently large language models (Qi *et al.* 2024, Pelletier *et al.* 2024) have been proposed in the HG literature. Despite significant advance, many of the existing HG approaches are designed for static settings and fail to leverage the temporal evolution of biomedical entities. This is problematic because the biomedical domain is highly dynamic with new facts emerging and old ones being obsolete every now and then. For example, consider the biomedical concept *Parkinson disease*. In the early 2000s, within the virology literature, its meaning used to be associated with concepts such as “Amyotrophic lateral sclerosis”, “Lysosomal storage diseases” and “Neoplasms”; however, in the 2020s it started to become associated with concepts like “Cytarabine” and “Cycloheximide”. Modeling such temporal evolution of biomedical concepts is crucial for generating hypotheses that are accurate and reflect up-to-date information landscape of the biomedical domain.

Recently, some approaches (Akujuobi *et al.* 2020a, 2020b, Zhou *et al.* 2022, 2024b, Jha *et al.* 2018) have proposed modeling the temporal change of biomedical concepts from the diachronic biomedical corpus. For example, Akujuobi *et al.* (2020a) models temporal dynamics using positive-unlabeled data, while Jha *et al.* (2018) encodes semantic drift through temporal matrix factorization. Nevertheless, no existing approach unifies spatial, temporal, and semantic evolution, even though integrating all three is critical for capturing the full scope of how biomedical concepts change over time.

Integrating these dimensions into a single coherent framework is fundamentally non-trivial. Each dimension requires distinctly different computational approaches: semantic modeling demands nuanced language understanding and representation, spatial modeling necessitates accurately learning structural graph representations, and spatio-temporal modeling requires capturing evolving patterns over time. Unifying these divergent computational strategies involves addressing significant methodological challenges, such as how best to align evolving semantic representations with temporal graph dynamics, efficiently encoding complex relational information, and ensuring seamless interaction among these diverse representations. To address this, we propose a novel method that captures the evolving nature of biomedical knowledge by modeling changes in a concept’s semantic meaning. Our approach is motivated by two key principles: the Distributional Hypothesis, which posits that word meanings emerge from the context of co-occurring concepts (Sahlgren 2008), and Conceptual Change (Posner *et al.* 1982, Treagust and Duit 2008), which describes how concepts evolve as they encounter new information. These principles guide us in tracking how biomedical concepts change meaning as they interact with new concepts over time.

A key contribution of our work is to adopt these principles to model the semantic, spatial, and temporal dimensions of biomedical concepts in a unified approach. We model the HG task as a sequence of graphlets which spans over discrete time steps. Each graphlet represents a snapshot of biomedical concepts and their connections with each other at discrete time steps. This enables us to track the evolution of spatial relationships between concepts over time, thereby guiding how our approach interprets changes in the meaning of these concepts. By modeling these dimensions in a unified manner, our approach provides a rich understanding of how biomedical concepts evolve. As a result, our approach generates more accurate predictions, yielding hypotheses with potential for scientific discovery.

The main contributions of our work can be summarized as follows:

- We propose a new approach to HG that exploits spatial, temporal, and semantic changes in biomedical concepts for accurate HG. This has immediate practical benefits for web-based literature mining tools designed for applications in life science and biomedicine.
- Unlike existing HG approaches that model spatial, temporal, and semantic changes independently, our proposed approach is the first to leverage them collaboratively in a unified framework. This holistic modeling enables our approach to learn feature representations that are not only more robust but also have superior predictive power, leading to more reliable and meaningful hypothesis generation.
- Extensive experiments on multiple datasets show that unifying spatial, temporal, and semantic changes in concepts with our proposed approach consistently outperforms baseline methods. Moreover, case studies show that the proposed approach generates hypotheses that are medically sensible and of potential interest to the domain experts.

## 2 Related works

Existing approaches in biomedical HG have predominantly emphasized modeling the semantic, spatial, or spatio-temporal dimensions independently, without integrating all three dimensions comprehensively. Below, we categorize existing methods and discuss their advancements and limitations.

**Semantic Methods.** Semantic approaches focus on representing biomedical concepts by capturing their meanings through embeddings derived from textual contexts. Traditional semantic methods rely on static representations to encode semantic context, facilitating tasks such as link prediction and literature-based discovery (Spangler *et al.* 2014, Srinivasan, 2004, Gopalakrishnan *et al.* 2019, Jha *et al.* 2019, Ding *et al.* 2024). These approaches typically utilize static statistical methods or co-occurrence measures to infer implicit associations. Recent advances in semantic modeling have leveraged transformer-based pre-trained language models (PLMs) such as BERT (Devlin, 2018) and BioBERT (Lee *et al.* 2020), significantly enhancing the semantic representation of biomedical text by capturing deep contextual meanings. These PLMs excel at nuanced semantic extraction, dramatically improving downstream tasks like biomedical link prediction and relation extraction (Yao *et al.* 2019). However, semantic methods are inherently limited due to their static nature. The semantic embeddings produced by PLMs like BioBERT do not evolve with new biomedical knowledge. Consequently, they cannot capture temporal shifts in the meanings of concepts as new contexts emerge over time, rendering them ineffective in dynamically evolving biomedical knowledge domains. This is a critical limitation for HG, as forecasting future associations requires accommodating changing conceptual semantics.

**Spatial Methods.** Spatial methods primarily involve representing biomedical concepts through structural relationships encoded within static graphs. Prior HG works in spatial modeling (Cameron *et al.* 2015, Pu *et al.* 2023) have focused on graph-based representations, utilizing concept co-occurrence and structural connectivity to identify potential novel relationships. Advanced spatial models such as Graph Convolutional Networks (GCNs) (Kipf and Welling, 2016), Graph Attention Networks (GATs) (Veličković *et al.* 2017), and Graph Transformers (Shi *et al.* 2020) have substantially improved graph-based representation learning. These models aggregate information from neighboring nodes and have shown promise in capturing complex structural patterns across diverse domains (Jafari *et al.* 2024, Li *et al.* 2023). Despite their strengths in modeling structural relationships, spatial methods face significant limitations. Primarily, they rely on static snapshots of knowledge, neglecting the temporal dynamics critical for understanding biomedical concept evolution. Without temporal context, these models cannot effectively predict emerging relationships as structural patterns evolve. Additionally, purely spatial methods fail to incorporate rich semantic contexts, limiting their ability to accurately capture nuanced biomedical relationships necessary for high-quality hypothesis generation.

**Spatio-Temporal Methods.** Spatio-temporal methods aim to overcome limitations of purely semantic or spatial methods by modeling both structural and temporal changes. These approaches integrate graph structures with temporal dynamics, enabling predictions of evolving relationships. Prior HG research employing spatio-temporal modeling (Akujuobi *et al.* 2020b, 2020a; Zhou *et al.*, 2022, 2024a, 2024b) has effectively captured temporal variations in structural connections. Notable general spatio-temporal frameworks include the Diffusion Convolutional Recurrent Neural Network (DCRNN) (Li *et al.* 2017) and Temporal Graph Network (TGN) (Rossi *et al.* 2020). DCRNN integrates graph convolutions with recurrent neural networks to model the temporal evolution of relationships, whereas TGN uses temporal memory states to dynamically update node interactions, significantly enhancing predictive capabilities. Spatio-temporal models lack explicit representation of semantic evolution. By focusing solely on structural relationships and temporal co-occurrences, these methods inherently discard detailed semantic context of concepts as they appear in the literature, crucial for nuanced hypothesis generation. Consequently, they struggle to accurately predict future relationships that rely heavily on evolving concept meanings, potentially leading to irrelevant or incorrect hypotheses.

## 3 Hypothesis generation problem

We formulate hypothesis generation as a temporal link prediction task on a dynamic biomedical concept graph. Our goal is to predict whether a link (i.e. co-occurrence) between two biomedical concepts will appear at a future time step by leveraging historical data mined from biomedical research publications. This involves identifying when two concepts, which may not have co-occurred previously, are likely to co-occur in future research, thus signaling a new relationship that can inspire further investigation or experimentation.

Inspired by recent HG methods (Akujuobi *et al.* 2020b, 2020a, Zhou *et al.* 2022, 2024a, 2024b), we represent this task using a dynamic graph, denoted as $G=\{G_{0},\ldots ,G_{t},\ldots ,G_{T}\}$, which spans discrete time steps t∈{0,…,T}, where *T* is the total number of time steps. Each graphlet $G_{t}=(V,E_{t})$ represents the state of biomedical knowledge at time step *t*. The set V={1,…,|V|} consists of a total number of |*V*| biomedical concepts, specifically Medical Subject Headings (MeSH) terms as defined by the National Library of Medicine (Leblanc *et al.* 2024). Each node i∈V corresponds to a unique biomedical concept, which remains constant across all time steps. The set of edges $E_{t}$ at time *t* captures the co-occurrences between concepts. An edge $(i,j)\in E_{t}$ indicates that concepts *i* and *j* co-occurred in at least one biomedical article within the time frame represented by *t*. At the initial time step (t=0), no co-occurrences are present, i.e., $E_{0}=\emptyset$. Formally, the HG task is to predict future co-occurrences between pairs of concepts (i,j) at time *T* using the historical graph information up to time T−1. By accurately predicting these co-occurrences, our model can uncover new and emerging relationships, leading to potentially novel hypotheses.

We construct dynamic graphs from PubMed articles by extracting concept co-occurrences using, PubTator3 (https://ftp.ncbi.nlm.nih.gov/pub/lu/PubTator3/), a state-of-the-art tool published by the National Institute of Health that outperforms BioBERT and GPT-4 for biomedical concept extraction (Wei *et al.* 2024). To minimize noise, we apply three filtering steps: (i) remove entries with missing concept IDs, (ii) restrict concepts to standardized MeSH terms only, and (iii) require concepts to co-occur within the same article to form edges, which filters potential cross-article noise. Additional construction details are provided in the Appendix.

## 4 Methodology

**Figure 1.**
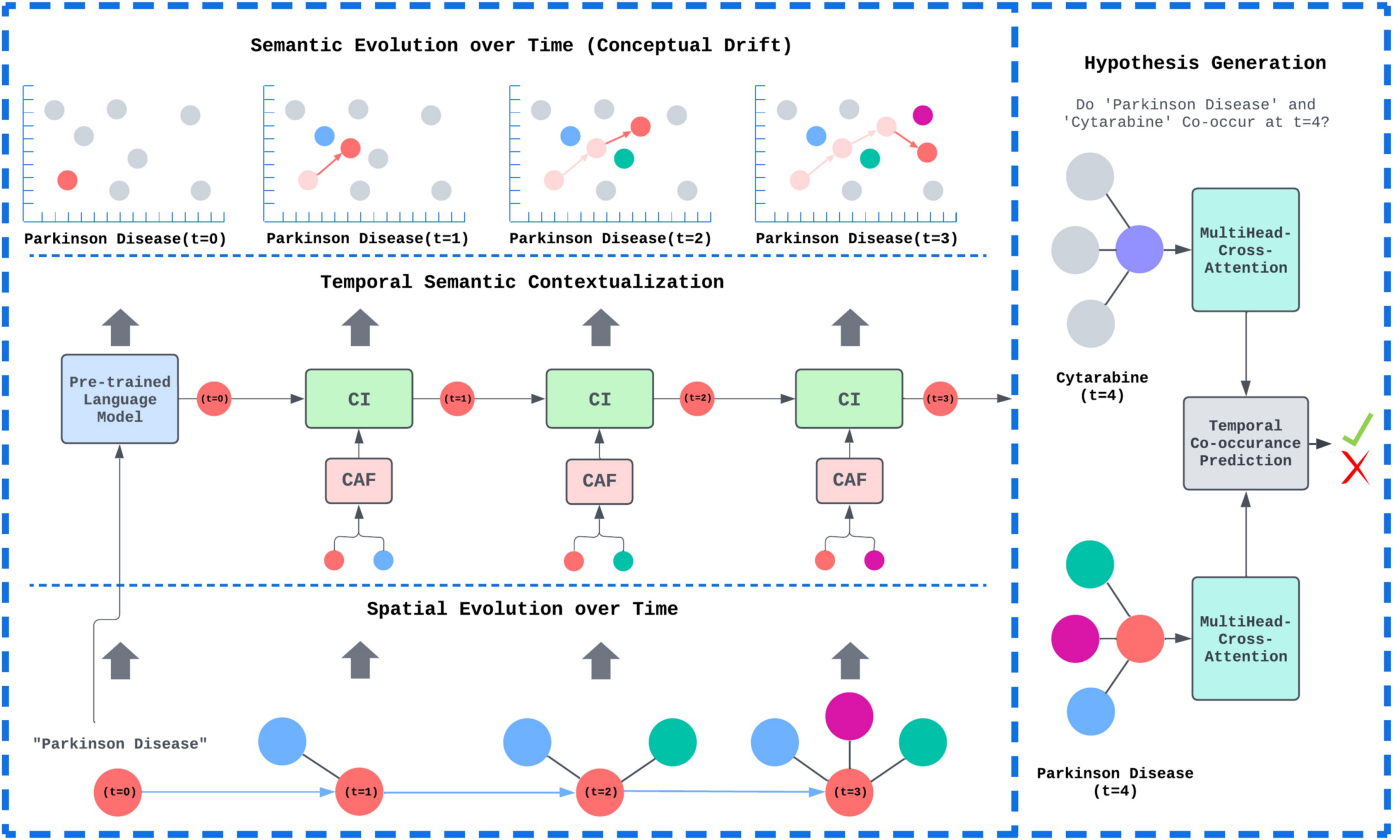
Overview of ConceptDrift. The bottom part of the figure shows the spatial evolution of “Parkinson Disease” in a temporal dynamic graph. The middle part of the figure depicts how the Temporal Semantic Contextualization (TSC) framework integrates “Parkinson Disease” with different concepts it co-occurs with over time to update its semantic state. The top part of the figure illustrates how TSC models conceptual drift (i.e., semantic evolution) over time. The right part of the figure demonstrates hypothesis generation through temporal co-occurrence prediction with Multi-head Cross Attention.

We introduce ConceptDrift, a unified approach that models how the meaning of concepts change over time by collaboratively integrating spatial, temporal, and semantic information. Unlike prior efforts that treat these separately, ConceptDrift unifies them with our novel framework called Temporal Semantic Contextualization (TSC), enabling accurate discovery of future co-occurrences that serve as potential hypotheses. By using TSC, ConceptDrift is able to accurately predict future co-occurrences between biomedical concepts and articulate the connection between the two concepts as a hypothesis with potential for scientific discovery. We illustrate an overview of how ConceptDrift models the change of meaning in biomedical concepts using TSC in Fig. 1.

### 4.1 Conceptual drift

Our approach is driven by two key principles: the Distributional Hypothesis and Conceptual Change as defined and discussed in the Introduction. The Distributional Hypothesis argues that the meaning of a biomedical concept can be inferred by the neighboring concepts it co-occurs with (Sahlgren 2008). Conceptual Change refers to the way people’s understanding of concepts can shift when exposed to new knowledge (Posner *et al.* 1982, Treagust and Duit, 2008). Building on these ideas, we introduce Conceptual Drift, which extends the Distributional Hypothesis to account for how the meanings of biomedical concepts evolve over time. Conceptual Drift states that the meaning of a concept changes when it begins to co-occur with new concepts.

For a given time *t*, we define the neighborhood of a concept *i* as the set of concepts that have co-occurred with *i* at least once from time step 1 up to time step *t*. Two biomedical concepts, *i* and *j*, are said to be similar at time *t* if their neighborhoods are similar, meaning their respective sets of co-occurring concepts overlap to a some extent. In the Appendix, we empirically validate conceptual drift occurring in real-world data by comparing the overlap of neighborhoods of co-occurring concepts with the neighborhoods of non-co-occurring concepts to see how much overlap similar neighborhoods have in practice. In the context of the hypothesis generation, this definition implies that we are predicting whether two concepts will co-occur at a future time step based on how their neighborhoods evolve dynamically over time.

### 4.2 Temporal semantic contextualization

We propose Temporal Semantic Contextualization (TSC), a framework for modeling Conceptual Drift by unifying the spatial, temporal, and semantic changes of biomedical concepts. In our TSC framework, each biomedical concept has a semantic state that will evolve over time. They are initialized with concept embeddings that reflect the concept’s semantics. Each time a concept co-occurs with another concept, the new context of the concept is captured and is integrated with the concept’s meaning to update its semantic state. Finally, before a prediction is made on whether two concepts will co-occur, special embeddings will be generated with temporal multi-head cross attention to enrich the concept’s representation with the temporal evolution of the concept’s neighborhood.

#### 4.2.1 Semantic states

We represent each biomedical concept using a semantic state that evolves over time. For a given concept *i* at a time *t*, its semantic state is denoted as $s_{i}(t)$. Semantic states are central to our framework’s integration of spatial, temporal, and semantic changes in concepts, as they capture how each concept’s meaning shifts through new co-occurrences. Specifically, as concepts interact and co-occur with others, their semantic states are updated to reflect changes in context.

To initialize these semantic states, we use a pre-trained language model (PLM) (Lee *et al.* 2020) since it can provide rich semantics for text embeddings. By inputting all the concepts in *V* into a PLM, we generate the concept embeddings for all concepts, resulting in a matrix $X\in {\mathbb{R}}^{|V|\times d}$, where *d* is the dimension of the embedding space. Each row in **X** corresponds to the concept embedding of a concept i∈V. At the initial time step (t=0), these embeddings are used to initialize the semantic state of each concept in the graph. Specifically, the semantic state $s_{i}(0)$ for concept *i* is set as its corresponding embedding from the matrix **X**. By grounding the initial state of each concept in its concept embedding, we provide the foundation for modeling how concepts change meaning over time as they interact with other concepts within the graph. In our implementation, we use BioBERT (Lee *et al.* 2020) since it is trained on a vast corpus of biomedical literature, which enables it to encapsulate the semantic nuances of biomedical concepts based on their contextual usage in scientific texts.

#### 4.2.2 Contextual integration

At a given time step *t*, the graphlet $G_{t}$ indicates when *i* co-occurs with another concept *j*, introducing new context for *i’*s evolving meaning. When this occurs, *i’*s previous semantic state must be updated to integrate the context. This contextual integration process involves two steps. First *i’*s new context must be captured. Then *i’*s semantic state is updated by integrating the captured context with *i’*s previous semantic state. To capture *i’*s new context, we propose the Context Aggregation Function (CAF). CAF aggregates the previous semantic state of concept *i*, $s_{i}(t_{p})$, where $t_{p}$ represents the last time step the semantic state of the concept was updated, with the previous semantic state of the new co-occurring concept *j*, $s_{j}(t_{p})$, and a time difference vector Δt (Kazemi *et al.* 2019). While prior spatio-temporal graph methods (Rossi *et al.* 2020, Wang *et al.* 2021b) use temporal encodings to weight structural recency, CAF leverages Δt to model semantic receptivity, i.e. how long a concept has maintained its current meaning determines how strongly new co-occurrences influence its semantic evolution. This is crucial in biomedicine where concepts like ‘Cytarabine’ undergo gradual semantic shifts as the field advances, requiring us to capture not just when interactions occur, but how temporal distance affects meaning change. By aggregating these three elements, we are able to combine the meaning of both concepts along with the recency of *i’*s interaction, allowing us to comprehensively capture *i’*s new context. CAF captures the new context as follows:


(1) $${\text{CAF}}_{i}(t)=s_{i}(t_{p})||s_{j}(t_{p})||\Delta t$$

where ${\text{CAF}}_{i}(t)$ is the aggregation of $s_{i}(t_{p})$, $s_{j}(t_{p})$, and Δt, and ‖ is the concatenation operator. Concatenation is used for aggregation since it preserves the semantic information from both concepts and can incorporate Δt without information loss.

Once *i’*s new context is captured, the new context must be integrated with *i’*s previous semantic state to create *i’*s new semantic state ($s_{i}(t)$). This step is termed Contextual Integration (CI). It is essential for modeling how the meaning of a concept evolves over time as its interactions change within the temporal graph. CI takes the captured context, as represented by ${\text{CAF}}_{i}(t)$, interprets what information from it is important, and updates *i’*s previous semantic state with new context. CI also inputs $s_{i}(t_{p})$ to preserve the historical meaning of concepts and avoid extreme shifts in concepts representations each time concepts interact with new concepts. CI integrates the new context and updates *i’*s semantic state as follows:


(2) $$s_{i}(t)=\text{CI}({\text{CAF}}_{i}(t),s_{i}(t_{p}))$$

In practice, CI can be any learnable function that can integrate new context while maintaining important historical information. In our experiments, we define CI to be a Gated Recurrent Unit (GRU) because it provides an effective balance between expressiveness and efficiency. GRUs are designed to capture long-term dependencies while avoiding the parameter overhead of more complex recurrent architectures such as LSTMs (Cho, 2014). While alternative architectures such as Transformer encoders or feed-forward networks could in principle be applied, the former introduces substantially higher computational cost, and the latter lacks the temporal gating needed to retain long-range contextual information.

### 4.3 Hypothesis generation

At a future time step t+1, we want to predict whether two concepts *i* and *j* will co-occur with each other. We first generate predictive embeddings out of the concept’s semantic states and their neighborhood of concepts using temporal multi-head cross attention. These embeddings are then used to predict whether a co-occurrence will exist between the two concepts. If a co-occurrence is predicted, it can be articulated as a potential hypothesis in natural language.

To predict future co-occurrences between any two concepts *i* and *j*, it is important to consider the relevant up-to-date semantic states of the concepts’ neighborhoods and when each neighboring concept first co-occurred with *i* or *j* in addition to *i* and *j*s current semantic state. Although a concept’s semantic state reflects past co-occurrences, neighboring concepts may update their semantic states over time, possibly having new information beneficial for prediction. We use temporal multi-head cross-attention to get a weighted sum of a concept’s neighbors based on their most recent semantic states and when they first interacted with the concept. It ensures that the model captures relevant information from the most recent representations of concepts and the temporal relevance of their interactions, leading to more accurate future predictions. The multi-head cross-attention mechanism introduces a query **q**, keys **K**, and values **V**. The query holds what information we want to focus on and is used to see which elements in list of potential matches are most relevant to it. The keys represent the potential matches. The values are the same as the keys, however, the keys relevancy with the query is used to output a weighted sum of the values. In our context, a concept *i* represents the query, while *i’*s neighborhood represents the keys and values. Multi-head cross-attention is used to weigh a concept *i’*s neighbors based on their semantic and temporal relevance to *i*. By taking the *i* and its neighbors’ most recent semantic states and concatenating them with temporal encodings (Kazemi *et al.* 2019), we accomplish this.

We define the query as the semantic state of concept *i* at time *t*, concatenated with a temporal encoding of the current time step:


(3) $$q=s_{i}(t)||\phi (t)$$

where ‖ denotes the concatenation operator, and ϕ(t) represents the temporal encoding of the current time step. We also define the keys and values as the most recent semantic states of *i’*s neighbors and concatenate them with temporal encodings of the time they first co-occurred with *i*:


(4) $$K=V=[s_{1}(t)||\phi (t_{1}),\ldots ,s_{N}(t)||\phi (t_{N})]$$

Here $t_{1},\ldots ,t_{N}$ denotes the time steps when each neighbor (1,…,N) first co-occurred with *i*. We use first co-occurrence times rather than recency based times because the evolving semantic states already capture recent interactions. First co-occurrence times provide a distinct signal marking potential discovery events, enabling the model to distinguish between established relationships and emerging ones.

The multi-head cross-attention inputs the query, keys and values and outputs a vector $h_{i}(t)$ that holds the most relevant temporal and semantic information in concept *i’*s neighborhood:


(5) $$h_{i}(t)=\text{MultiHeadCrossAttention}(q,K,V)$$

To integrate this information with the concept’s current semantic state, we concatenate $h_{i}(t)$ with $s_{i}(t)$ and pass the result through a Multi-Layer Perceptron (MLP) to produce the predictive embedding $z_{i}(t)$:


(6) $$z_{i}(t)=\text{MLP}(s_{i}(t)||h_{i}(t))$$

The predictive embedding has a concept’s current semantic state with relevant information from the up-to-date semantic states of the concept’s neighborhood and the time of its past co-occurrences, leading it to produce more accurate predictions.

After generating the concepts *i* and *j’*s predictive embeddings $z_{i}(t)$ and $z_{j}(t)$, we compute the probability of whether *i* and *j* will co-occur at a future time step t+1. The probability is denoted as p(i,j|t+1). To do this we pass the predictive embeddings through an MLP and use sigmoid function to compute the probability of co-occurrence:


(7) $$p(i,j|t+1)=\sigma (\text{MLP}(z_{i}(t)+z_{j}(t)))$$

where σ(·) is the sigmoid function.

## 5 Results

**Datasets.** Following standard hypothesis generation practices (Akujuobi *et al.* 2020a, 2020b, Zhou *et al.* 2022, 2024a, 2024b), we train models on historical PubMed data and evaluate their ability to rediscover future publications. We construct dynamic graph datasets from PubMed articles in Virology, Neurology, and Immunology spanning 2000–2024, with each year as a separate time step, similar to Tyagin and Safro (2024)’s approach. This annual resolution improves upon prior work using multi-year periods (Akujuobi *et al.* 2020a, 2020b, Zhou *et al.* 2022, 2024a, 2024b). Further, ConceptDrift’s architecture supports finer temporal granularities since computational complexity depends on edge events rather than time steps. We train on data through 2022, use 2023 for validation, and test on 2024. To prevent data leakage, we remove all 2024 test points that appeared in earlier time steps. Dataset statistics are shown in Table 1, with construction details in the Appendix.

**Table 1.** Statistics of the *Virology*, *Neurology*, and *Immunology* datasets.

Metric	Virology	Neurology	Immunology
Num. Concepts	7,254	10,848	15,058
Co-oc. 2000–2022	2,770,144	10,576,804	13,789,490
Co-oc. 2023	496,724	1,530,890	2,175,560
Co-oc. 2024	924	406	15,058

**Experimental Setup.** To evaluate the efficacy of ConceptDrift, we compare the performance of ConceptDrift on predicting co-occurrences in 2024 with a variety of baselines. We evaluate each method’s performance using metrics that indicate how well the methods can discriminate between true and false future co-occurrences while balancing precision and recall. When training and evaluating each method, we generate a 1:1 ratio of negative (false) concept co-occurrences by uniform sampling. Following established retrospective validation protocols (Akujuobi *et al.* 2020a, 2020b, Zhou *et al.* 2022, Tyagin and Safro 2024, Zhou *et al.* 2020a, 2024b), we train on historical literature and test on held-out future publications. To ensure the reliability of our findings, we ran ConceptDrift and TGN (with PLM features) 10 times each, reporting mean and standard deviation. Statistical significance is assessed using a two-sample t-test.

**Baselines and Metrics.** Prior HG methods typically fall into three categories: semantic, spatial, and spatio-temporal. For fair comparison, we select strong baselines from each category. We evaluate BERT (Devlin 2018) and BioBERT (Lee *et al.* 2020), two widely used PLMs known for their effectiveness in biomedical semantic representation and link prediction (Yao *et al.* 2019). We additionally evaluate ClinicalBERT (Huang *et al.* 2019), BiomedNLP (Gu *et al.* 2020) and SciBERT (Beltagy *et al.* 2019). These models provide a strong benchmark for purely semantic approaches. For structural modeling, we include GCN (Kipf and Welling 2016), GAT (Veličković *et al.* 2017), and Graph Transformer (Shi *et al.* 2020), which are powerful GNNs commonly used across different domains and tasks to learn static graph structures. We experiment against DCRNN (Li *et al.* 2017) and TGN (Rossi *et al.* 2020), two advanced models for dynamic graph representation, which effectively capture structural changes over time. We also evaluate STHG (Zhou *et al.* 2024b), a state-of-the-art HG method that outperforms other prevalent HG works (Akujuobi *et al.* 2020b, Zhou *et al.* 2022). Further details about each baseline we use are provided in the Appendix.

We use Area Under the ROC Curve (AUC) and Average Precision (AP) to evaluate predictive performance. AUC measures the model’s ability to rank true co-occurrences higher than false ones, while AP assesses the balance between precision and recall. Together, these metrics offer a robust evaluation of hypothesis prediction accuracy.

### 5.1 Main results

Our results, reported in Table 2, show that ConceptDrift consistently outperforms all baselines across all three datasets, underscoring how simultaneously modeling semantic, spatial, and temporal evolution yields stronger predictive accuracy than treating these dimensions in isolation. The results from 10 independent runs demonstrate that ConceptDrift achieves a higher mean AUC and AP across all PLMs and datasets, and the low standard deviation indicates the stability and reliability of our model’s performance. To ensure ConceptDrift’s superiority is not by chance, we compare ConceptDrift with 10 independent runs of TGN (with PLM embeddings as features) and see ConceptDrift outperforms TGN. In the Table 3, we report two-sample t-tests that shows these improvements are statistically significant.

**Table 2.** Main experiment results showing AUC and AP performance of models on three biomedical domains. Dimensions modeled are semantic (◯), spatial (□), and temporal (△).

Method	Dim.	Virology		Neurology		Immunology	
		AUC ↑	AP ↑	AUC ↑	AP ↑	AUC ↑	AP ↑
BERT	◯	0.663	0.666	0.823	0.765	0.764	0.745
BioBERT	◯	0.666	0.671	0.818	0.799	0.766	0.748
ClinicalBERT	◯	0.500	0.500	0.644	0.646	0.649	0.630
BiomedNLP	◯	0.784	0.754	0.783	0.754	0.792	0.785
SciBERT	◯	0.525	0.539	0.708	0.720	0.803	0.785
GCN	□	0.520	0.459	0.859	0.819	0.661	0.702
GAT	□	0.613	0.546	0.662	0.655	0.514	0.467
Graph Transformer	□	0.508	0.467	0.772	0.678	0.706	0.696
DCRNN	□△	0.542	0.656	0.827	0.797	0.702	0.758
STHG	□△	0.712	0.730	0.867	0.836	0.794	0.776
TGN	□△	0.839	0.816	0.881	0.866	0.855	0.818
TGN + BioBert	◯ □△	0.803±0.022	0.767±0.034	0.717±0.025	0.684±0.034	0.843±0.018	0.816±0.023
TGN + ClinicalBERT	◯ □△	0.812±0.021	0.778±0.027	0.719±0.025	0.686±0.036	0.834±0.019	0.803±0.026
TGN + BiomedNLP	◯ □△	0.830±0.016	0.808±0.019	0.721±0.028	0.685±0.028	0.809±0.034	0.771±0.044
TGN + SciBERT	◯ □△	0.795±0.023	0.763±0.027	0.712±0.030	0.674±0.029	0.845±0.018	0.820±0.024
ConceptDrift + BioBert	◯ □△	0.878±0.014	0.856±0.015	0.917±0.014	0.895±0.018	0.885±0.010	0.870±0.009
ConceptDrift + ClinicalBERT	◯ □△	0.877±0.009	0.856±0.010	0.900±0.012	0.876±0.010	0.891±0.012	0.874±0.010
ConceptDrift + BiomedNLP	◯ □△	**0.880** ± **0.008**	**0.859** ± **0.007**	0.894±0.010	0.880±0.010	0.894±0.010	0.880±0.010
ConceptDrift + SciBERT	°□△	0.872±0.013	0.852±0.013	**0.924** ± **0.011**	**0.908** ± **0.010**	**0.899** ± **0.012**	**0.883** ± **0.012**

**Table 3.** Two sample T-test between ConceptDrift and TGN results.

Dataset	Metric	T-statistic	*P*-value
Virology	AP	14.468646	$2.37\times 10^{-19}$
Virology	AUC	15.907414	$2.36\times 10^{-22}$
Neurology	AP	37.401726	$2.69\times 10^{-45}$
Neurology	AUC	41.617017	$1.15\times 10^{-47}$
Immunology	AP	12.768318	$7.89\times 10^{-17}$
Immunology	AUC	12.910813	$4.61\times 10^{-18}$

By modeling the spatial, temporal, and semantic dimensions in an integrated manner, ConceptDrift captures the evolving relationships and contextual meaning of biomedical concepts, which is critical for accurately predicting future co-occurrences. In contrast, baselines that only leverage one or two dimensions fall short, highlighting the importance of a unified approach. ConceptDrift performs robustly across different PLMs. These results demonstrate the model’s effectiveness and generalizability, independent of any specific PLM. ConceptDrift also demonstrates robustness in performance even with smaller training samples. The *Virology* data set has under 3 million co-occurrences from 2000–2020, compared to over 10 million for *Neurology* and *Immunology*. Despite the smaller training data, ConceptDrift gets the best AUC and AP of 0.880 and 0.859, respectively. This is due to its ability to model Conceptual Drift, which captures how biomedical concepts evolve over time. By dynamically updating concept meanings and generating temporal embeddings, ConceptDrift can accurately predict future co-occurrences, even with limited data.

ConceptDrift’s consistent outperformance across datasets suggests that modeling conceptual drift helps filter spurious associations. By requiring concepts to maintain evolving semantic relationships through their neighborhood dynamics over multiple time steps, our approach implicitly favors stable, meaningful connections over transient co-occurrences that may arise from publication artifacts or trending topics.

### 5.2 Ablation study

In this section we analyze the contribution of key components in ConceptDrift and subsequently discuss how each dimension (semantic, spatial, and temporal) collectively drives our performance. Our results are reported in Table 4.

**Table 4.** Ablation study results for ConceptDrift when removing semantic initialization (sem. init.), contextual integration (cont. integ.), refeeding sematic states (refeeding $s_{i}(t_{p})$), or temporal multi-head cross-attention (temp. cross-attn).

Method	2024 AUC ↑	2024 AP ↑
*Virology*		
ConceptDrift	0.847	0.830
- w/o sem. init.	0.807	0.805
- w/o cont. integ.	0.738	0.737
- w/o refeeding $s_{i}(t_{p})$	0.832	0.801
- w/o temp. cross-attn.	0.728	0.706
*Neurology*		
ConceptDrift	0.900	0.884
- w/o sem. init.	0.755	0.726
- w/o cont. integ.	0.772	0.729
- w/o refeeding $s_{i}(t_{p})$	0.779	0.817
- w/o temp. cross-attn.	0.778	0.760
*Immunology*		
ConceptDrift	0.907	0.891
- w/o sem. init.	0.792	0.790
- w/o cont. integ.	0.781	0.764
- w/o refeeding $s_{i}(t_{p})$	0.880	0.817
- w/o temp. cross-attn.	0.788	0.784

First, we assess the impact of semantic initialization by removing the initialization of embeddings for concepts. Table 4 shows that performance deteriorates on all three datasets without semantic initialization, highlighting the synergy of the semantic dimension with spatial and temporal evolution of concepts within the datasets. In this ablation, ConceptDrift lacks a semantic foundation and thus can only capture spatial and temporal changes without grounding concept representations in rich semantic features.

Second, we examine how updating semantic states as concepts interact affects performance by ablating the contextual integration process. This removal yields a substantial drop on all three datasets, underscoring the need for a collaborative approach to unify the spatial, temporal, and semantic dimensions. Simply incorporating each dimension in a naive manner is insufficient for capturing the evolving nature of biomedical concepts.

Third, we examine the effectiveness of our design choice for the contextual integration module (Equation 2) that inputs both the aggregated context (Equation 1) and previous previous semantic state $s_{i}(t_{p})$, which is already in aggregated context. We do this by modifying the the contextual integration module to only input the aggregated context. This modification results in a slight drop in performance indicating the importance of feeding the $s_{i}(t_{p})$ as an additional input. Altogether, this strengthens the signals of past representation of concepts to the contextual integration module, helping the historical meaning of concepts while still incorporating new information about the concepts’ evolving contexts.

Finally, we intend to assess how well temporal multi-head cross-attention improves ConceptDrift by using predictive embeddings that enrich a concept with relevant information from the up-to-date semantic states of the concept’s neighborhood and the time of its past co-occurrences. To test this, we remove temporal multi-head cross-attention and rely solely on the semantic states for prediction. Similar to the semantic initialization ablation, we observe that temporal multi-head cross-attention is essential for maintaining high performance. These results highlight the critical importance and non-trivial nature of our methodological integration. Removing key integrated components, such as semantic initialization or temporal cross-attention, substantially degrades predictive performance, underscoring how each dimension interacts synergistically within ConceptDrift.

### 5.3 Data scarcity robustness

**Figure 2.**
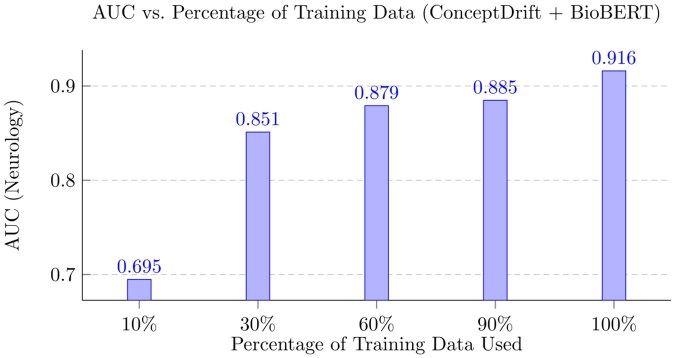
AUC after training on different percentages of the Neurology training dataset.

To demonstrate ConceptDrift’s robustness to low-data scenarios and its strong generalization ability, we experiment how well ConceptDrift performs when trained on randomly sampled smaller proportions of training data. We report the performance of ConceptDrift (with BioBERT concept embeddings) when trained on 10%, 30%, 60%, 90%, 100% of the co-occurances from the Neurology training data and report the results in Fig. 2. We see that as fraction of training data decreases, the performance of ConceptDrift gracefully decreases. In particular, even with only 30% of the training data, ConceptDrift is able to achieve a reasonable AUC of 0.851, outperforming many of its baselines trained on the entire dataset. This demonstrates ConceptDrift’s strong generalization capabilities and robustness to low-data settings.

### 5.4 Conceptual drift analysis

**Figure 3.**
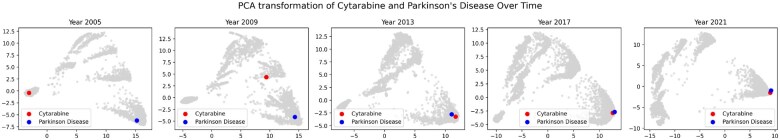
PCA transformations of Cytarabine and Parkinson’s Disease semantic states after ConceptDrift trains on Neurology Dataset for several years.

We tracked the semantic trajectories of Cytarabine and Parkinson Disease using the neurology dataset, analyzing how their semantic state embeddings evolved toward their 2024 real-world connection (Li *et al.* 2024). Figure 3 shows these concepts’ semantic states occupied distinct semantic regions from 2005–2009, then Cytarabine reached the same cluster of concepts that Parkinson Disease occupied by 2013. By 2021, the concepts had converged in the embedding space, foreshadowing their 2024 co-occurrence in literature. This temporal evolution demonstrates how ConceptDrift’s Temporal Semantic Contextualization mechanism anticipates novel biomedical connections by tracking dynamic concept meanings through evolving neighborhood contexts.

**Figure 4.**
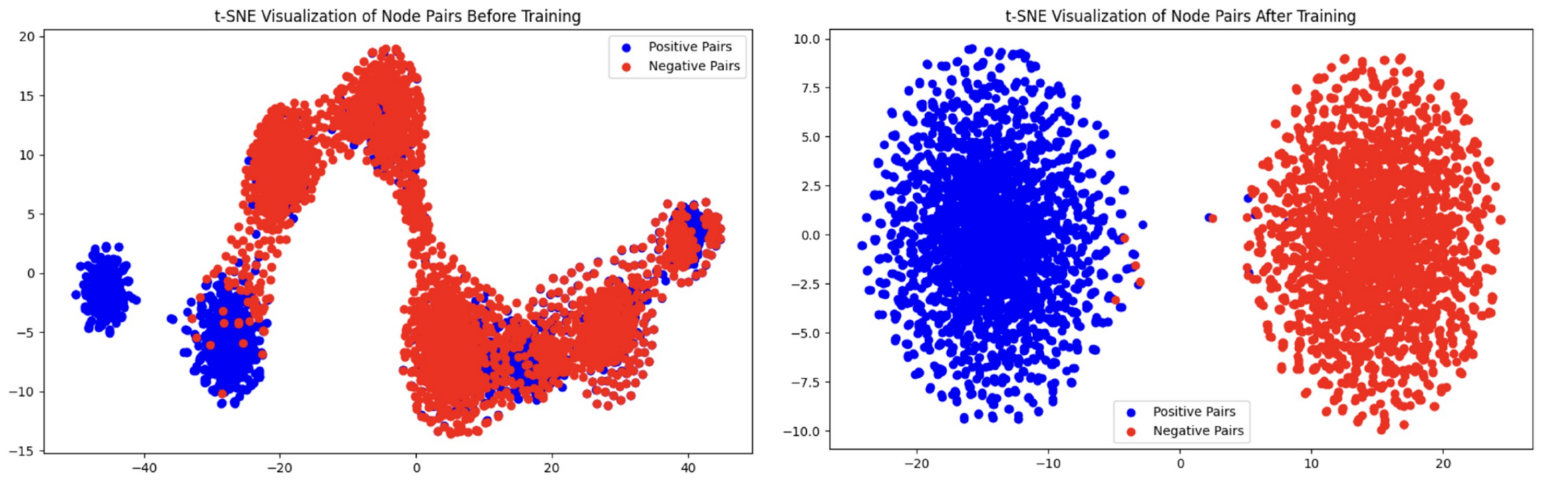
t-SNE embeddings of ConceptDrift’s last hidden layer for unseen Virology 2023 positive and negative co-occurrence concept pairs before (left) and after (right) training.

We also observe t-SNE (Van der Maaten and Hinton, 2008) projections of ConceptDrift’s final hidden layer (Fig. 4) and see a clear discrimination between positive and negative co-occurrences on unseen data, confirming the model captures dynamic semantic changes. This discrimination may reflect how temporal evolution filters spurious associations. Concepts must maintain evolving semantic relationships through neighborhood dynamics over multiple time steps, potentially favoring persistent co-occurrences that correlate with meaningful biological relationships rather than incidental mentions. In the appendix we provide more analysis on conceptual drift.

### 5.5 Scalability

The computational cost of ConceptDrift is dominated by two components: contextual integration via GRU updates for each observed co-occurrence and temporal multi-head cross-attention during prediction. For contextual integration, each edge event incurs $O(d^{2})$ operations per GRU update, where *d* is the hidden dimension. For prediction, the cross-attention mechanism requires $O(d^{2})$ operations for linear projections and $O(H\cdot \bar{N}\cdot d)$ for computing attention over $\bar{N}$ average neighbors with *H* heads. With |*E*| edge events processed during training, the overall complexity is $O(|E|\cdot d^{2}+H\cdot |E|\cdot d)$, which simplifies to $O(|E|\cdot d^{2})$ as |*E*| dominates. Empirically, ConceptDrift demonstrates near-linear scalability with respect to the number of edges. On the Virology dataset, the model trains within one and half hours on a single NVIDIA A6000 GPU, while Neurology and Immunology require less than two and three hours, respectively. These results confirm that ConceptDrift can efficiently scale to tens of millions of temporal co-occurrences while maintaining state-of-the-art predictive performance.

## 6 Conclusion

We introduced ConceptDrift, a framework that unifies semantic, spatial, and temporal dimensions for biomedical hypothesis generation. By modeling Conceptual Drift through evolving neighborhood dynamics, ConceptDrift captures how concept meanings shift over time. Experiments on multiple datasets show consistent outperformance of baseline methods, underscoring the value of integrating all three dimensions.

## Data Availability

The data used for our experiments are available on Figshare at https://doi.org/10.6084/m9.figshare.29975476.v1.
